# The effect of statins on the organs: similar or contradictory?

**DOI:** 10.15171/jcvtr.2017.11

**Published:** 2017-06-29

**Authors:** Yasin Ahmadi, Amir Ghorbanihaghjo, Hassan Argani

**Affiliations:** ^1^Student Research Committee, Tabriz University of Medical Sciences, Tabriz, Iran; ^2^Biotechnology Research Center, Tabriz University of Medical Sciences, Tabriz, Iran; ^3^Drug Applied Research Center, Tabriz University of Medical Sciences, Tabriz, Iran

**Keywords:** Adipose Tissue, Cholesterol, HDL-C, Ileum, Statins

## Abstract

Hydroxy-Methyl-Glutaryl-CoA reductase (HMGCR) – the main enzyme of the cholesterol biosynthesis pathway – is mostly inhibited by statins in hepatocytes. In spite of the other tissues, liver utilizes cholesterol in different ways such as the synthesis of bile acids, excretion in to the intestine and synthesis of lipoproteins. Therefore, statins theoretically alter these pathways; although, there have not been such effects. In this review, we aim to show the roles of extra-hepatic tissues, in particular intestine, adipose and cutaneous tissues in providing the cholesterol after reduction of the whole body cholesterol content by statins.

## Introduction

Cholesterol absorption in the small intestine and cholesterol synthesis in hepatic and extra-hepatic tissues contribute to maintaining cholesterol homeostasis in the body. Our body loses at least 900 mg cholesterol daily and this must be compensated through diet or de novo synthesis in the body.^1^

## Cholesterol metabolism in the liver

Not only dietary cholesterol is directly transported to the liver, hepatocytes also synthesize about 60%-70% of the whole body cholesterol.^2^

Cholesterol is precursor to bile acids in the liver, steroid hormones in steroidogenic organs and Vitamin-D in subcutaneous tissue.^3^ Regarding the vital roles of cholesterol, inhibition of the cholesterol biosynthesis by statins without providing cholesterol for liver may result in inefficiency of liver.

Induction of reverse cholesterol transport (RCT) process which transfers the excess amounts of cholesterol from extra-hepatic tissues to liver and also increase in the cholesterol absorption level in the intestine can be considered as the main ways of compensation of the cholesterol insufficiency in the liver at the result of statin therapy.

Except for liver, some tissues and cells have a remarkable role in cholesterol metabolism; these include gastro-intestinal tract, adipose tissue and macrophages. The role of these tissues and cells in cholesterol metabolism have been shown in the next.

## Statins induce the component of RCT

Since there is a permanent cholesterol exchange between serum and tissues, the cholesterol of different tissues originates either from *in situ* synthesis or serum.^4^ Therefore, serum and other tissues can be considered as cholesterol sources for liver and vice versa. Peripheral cells (apart from steroidogenic and skin cells) cannot degrade cholesterol and so the excess amount of cholesterol in these cells must be exported to liver.^5^ Most of studies have emerged that statins induce different components of RCT process including the ATP binding cassette transporter-A1 (ABCA1), ATP binding cassette transporter-G1 (ABCG1), scavenger receptor Class B type I (SR-BI) and Apo-AI (see Table 1).

**Table 1:** Effects of different statins on the components of reverse cholesterol transport and LDL metabolism in the liver

**Substance**	**Statin types**	**Effects**
Apo-A1	Pitavastatin, Atorvastatin Simvastatin,^6,7^ Cerivastatin^8^	Increase
ABCA1	Atorvastatin,^9^ Pitavastatin, Simvastatin^7^	Increase
HDL-C	Simvastatin,^7^ Pitavastatin, Lovastatin^10^	Increase
Apo-B100	Atorvastatin, Simvastatin^6^	Decrease
LDL-C	Atorvastatin, Simvastatin, lovastatin^6,7^	Decrease
LDL-R	Lovastatin, Simvastatin, Mevastatin (Compactin)^11^	Increase
LCAT	Simvastatin^7^	Increase

Abbreviations: Apo-A1: Apolipoprotein-A1, ABCA1: ATP-binding cassette transporter A1, HDL-C: high density lipoprotein cholesterol, Apo-B100: Apolipoprotein-B100, LDL-C: low density lipoprotein-cholesterol, LDL-R: low density lipoprotein-receptor, LCAT: lecithin cholesterol acyl transferase

In addition to induction of the RCT component, statins up-regulate expression of LDL-R on hepatocytes and so reduce the LDL-C in circulation. This effect of statins upon LDL-R can be considered as a feedback response by hepatocytes regarding the reduced hepatic cholesterol.

## The macrophage roles in RCT and statins effect on the macrophage cholesterol efflux

Macrophages are the major scavenger of oxidized LDL-C (ox-LDL) in the body.^12^ In spite of other extra-hepatic tissues, most of studies have shown the negative effects of statins on the ABCAI and ABCG1 expression in cultured macrophages. The effect of statin on these proteins in macrophages depends on the level of cholesterol loaded in the macrophages (see Table 2).^8^ It has been shown that statins in cholesterol depleted macrophage down-regulate ABCA1; however, in cholesterol loaded macrophage up-regulate the ABCA1 and ABCG1expression.^13^ Statins through reduction of oxysterols which are the natural ligand for liver X receptor alpha (LXRα) -LXRs are transcription factors- down-regulate the ABCA1 and ABCG1 expression as the downstream target of LXR in the macrophages.

**Table 2:** Effects of different statins on some components of reverse cholesterol transport and in the macro-phage

**Substance**	**Cholesterol load status**	**Statin types**	**Effects**
Cholesterol efflux to Apo-A1	Loaded,^9,11^* non-loaded ^11,13^	Atorvastatin,^9,13^ Compactin^11^	Increase,^9^ Decrease^9,11,13^ Without effect^11^*
ABCA1	Loaded,^9^ non-loaded ^8,11^	Atorvastatin^8,9,13,14^, Compactin^11^	Increase^9^, decrease ^11^
ABCG1	Loaded,^9^ non-loaded ^8^	Atorvastatin,^8,9,14^ Statins,^14^ Simvastatin,^7^ Compactin^11^	Increase^8,9,11,14^, Decrease^8^

Abbreviations: ABCA1: ATP-binding cassette transporter A1, ABCG1: ATP-binding cassette transporter G1.

On the other hand, a recent study showed that ABCA1 pathway in peripheral macrophages made an unremarkable contribution to HDL cholesterol loading process; it demonstrates that macrophages and other hematopoietically derived cells are not remarkably involved in lipidation of Apo-A1 via ABCA1.^15^ Also, mice lacking macrophage ABCA1 have no significant change in their plasma lipid profile. These findings show that macrophage ABCA1 plays an important role in RCT, however, the considerable amount of cholesterol efflux still occurs even in the absence of macrophage.^16^

Thus, macrophage ABCA1 does not have a major impact on the cholesterol content of HDL and liver ABCA1 plays the leading role in this regard.^15^ This means that even in the absence of statin therapy, macrophages have no a significant role in the cholesterol loading of HDL particles and subsequently RCT.

However, macrophages play an indispensable role in scavenging of ox-LDL^17^; the scavenger receptors most clearly linked to the ox-LDL uptake are Scavenger Receptor A (SR-A) and CD 36^12^ and these are responsible for 75%–90% of degradation of the acetylated/oxidized LDL.^18^ It has been demonstrated that CD36 is the major scavenger receptor for oxidized-lipoproteins.^19^ A feed-forward loop has been emerged; the higher taking levels of ox-LDL by macrophages results in the further activation of peroxisome proliferator-activated receptor-γ (PPAR-γ) which as a transcription factor perpetuates CD36 expression in spite of down-regulation of SR-AI/II; and thus potentially induces the ox-LDL uptake by macrophages.^19^ This means that macrophages internalize modified LDL through scavenger receptors. In spite of LDL-R, scavenger receptors are not reduced by the high intracellular cholesterol content. On the contrary, macrophage exposure to the high levels of ox-LDL results in up-regulation of the scavenger receptors. Hence, macrophages incubated continuously with ox-LDL are converted into foam cells.^12^

Regarding these evidences, macrophages act as an oxidized-cholesterol scavenger but not cholesterol synthesizer; thus, the main role of macrophages in cholesterol metabolism is because of the ox-LDL scavenging.

## Hepatic and intestinal Apo-A1 and ABCA1 as the main components of RCT and targets of the statins

The liver and intestine ABCA1-knockout mice have the reduced levels of HDL-C by 80% and 30% respectively; this finding indicates these organs as the sources of Apo-AI synthesis, are also the main organs responsible for lapidating newly synthesized lipid-poor Apo-AI via ABCA1-mediated lipid efflux.^16^ liver and intestine have been shown to be responsible for 90%-97% of the [^14^C] acetate incorporated into DPS which is measurable in all tissues.^20^

ABCA1 regulates the rate-limiting step in cellular cholesterol efflux to Apo-AI; ABCG1 conducts the cholesterol efflux from macrophages to HDL-C particles.^9^ The macrophages ABCA1 efflux cholesterol to lipid-poor Apo-AI, but not HDL, whereas, ABCG1 and SR-BI efflux cholesterol to HDL, but not Apo-AI.^21^

It has been shown that the infusion of recombinant pro Apo-AI enhances the fecal excretion of bile acids derivatives.^22^ Apo-AI increases the production of HDL in serum and subsequently induce RCT process^23^ and finally provides the required cholesterol for synthesis of bile acids in the liver.^24^ Statins through induction of the Apo-AI (Table 1) induce the RCT.

**Figure 1:**
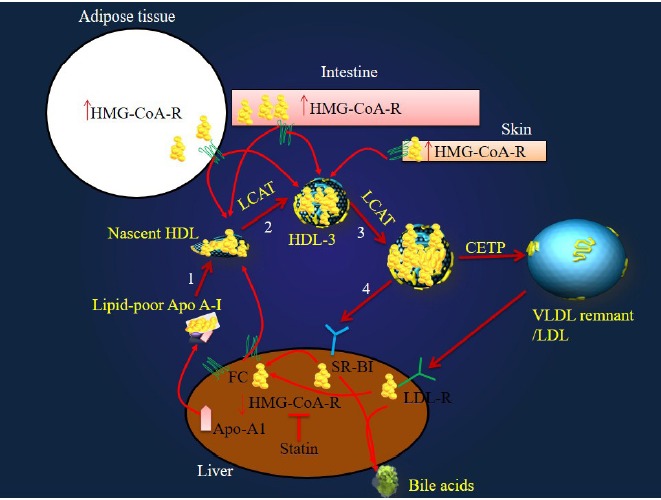
Statins induce expression of cholesterol transporters such as ABCA1, ABCG1 and SR-BI in the liver and extra-hepatic tissues such as intestine, adipose tissue and skin. Also, statins induce expression of Apo-AI in the hepatocytes. The increased expression of ABCA1, ABCG1 and SR-B1 leads to higher cholesterol transferring to Apo-AI, it results in higher production of HDL particles and so increases RCT (step 1-5). Because of the up-regulation of the cholesterol transporters, cholesterol does not accumulate in extra-hepatic tissues and so the inhibitory feedback of cholesterol on HMGCR is removed. Therefore, in extra-hepatic tissues de novo biosynthesis of cholesterol increases so that it can provide the required cholesterol for bile acids synthesis in the liver.

Abbreviations: ABCA1: ATP-Binding Cassette Transporter-A1, Apo-AI: Apolipoprotein-A1, Apo-B100: Apolipoprotein-B100, FC: Free Cholesterol, HDL-C: High Density Lipoprotein-Cholesterol, HMGCR: Hydroxy-Methyl-Glutaryl-CoA-Reductase, LCAT: Lecithin Cholesterol Acyl Transferase, LDL-R: Low Density Lipoprotein-Receptor, SR-B1: Scavenger Receptor-B1 VLDL: Very Low Density Lipoprotein.

In general, statins through up-regulation of transporters such as ABCA1 and ABCG1 increase the cholesterol load of HDL-particles by 5% to 15%^25,26^ which are finally transported to the liver (Figure 1). Thus statins through up-regulation of both transporters and Apo-A1 induce RCT process.

## Gain of the potential of over-production of cholesterol by extra-hepatic tissues after statins therapy

About 70% of the LDL-R is concentrated in the liver.^5^ Statins induce further expression of LDL-R on hepatocytes, thus induce receptor-mediated uptake of LDL-C and provide additional cholesterol for hepatocytes.^27,28^ Reverse cholesterol transport can be considered as a cholesterol source for liver; however, it can not completely compensate for the reduced *de novo* biosynthesis of cholesterol by statins in the liver; cholesterol biosynthesis in extra-hepatic tissues is only sufficient for normal functioning of those specific tissues.^29^ Therefore it is not clear how extra-hepatic tissues can provide such amounts of cholesterol for the liver, whereas these tissues were dependent on the hepatic derived cholesterol before statin therapy.

Statins increase the number of HDL-C particles in circulation^10^ and also induce the ABCA1 and ABCG1 expression which are the main cholesterol transporters.^8,30^ Therefore, statins enhance the cholesterol load of HDL^7^ and also enhance the reverse cholesterol transport which provides cholesterol for physiologic roles of liver.

RCT reduces the cellular cholesterol content and also down-regulates the cholesterol utilization process.^31^ Regarding the effect of statins on the liver and extra-hepatic tissues, it seems statins may decrease the cholesterol pool (but not cholesterol biosynthesis) in extra-hepatic tissues through different mechanisms; First, statins up-regulate LDL-R on hepatocytes^27,28^ and subsequently induce the rendering of additional LDL-C to liver instead of extra-hepatic tissues; furthermore, statins increase the number of HDL particles.^30^

Cholesterol imposes negative feedback on HMGCR at the transcriptional and translational levels and also induce degradation of HMGCR. ^32^ When the cellular cholesterol level is low, its inhibitory feedback effect on HMGCR is eliminated.^33^ On the other hand, because of the rapid transfer of cholesterol to HDL particles, cholesterol does not accumulate within the cells and the inhibitory feedback on HMG-CoA is removed. Thus, upon statin therapy the extra-hepatic tissues can gain the ability to compensate 900 mg cholesterol loss per day.

In man and other primate species the intestinal tract has a remarkable cholesterol synthesis ability,^34,35^ so that intestine can synthesis cholesterol as much as liver.^36^

Fallowing the cholesterol synthesis inhibition in the liver, small and large bowel and skin increase their cholesterol synthesis considerably, so that gastrointestinal tract and skin accounts for 39% and 44% of the total cholesterol synthesis respectively. However, epithelial barrier of the skin and continuous desquamation of hair, epithelial cells and sebum reduce the role of skin in providing circulatory cholesterol.^35^ Although, the liver and intestine ABCA1 transporters are vital for lapidating of lipid-poor Apo-AI, further cholesterol transferring and stabilizing of HDL are conducted by other tissues such as adipose tissue -the major pool of free cholesterol in the body.^16^ The role of different organs in cholesterol biosynthesis has been shown in Table 3.

**Table 3:** Role of different tissues in cholesterol biosynthesis

**Organ**	**% Cholesterol/sterol synthesis**
Intestine	60-70 circulatory cholesterol in subjects with a high cholesterol diet.^36^ Ileum has ability of cholesterol synthesis equal to 65% of cholesterol synthesis in the liver.^37^ With marked suppression of hepatic cholesterologenesis, small and large bowel account for 31% of the total sterol synthesis.^35^
Liver	54%^20^
Skin	With marked suppression of hepatic cholesterologenesis, skin accounts for 44% the total sterol synthesis.^35^
Liver+ astrointestinal	90% of whole body cholesterol synthesis^37^ 90%-97% of the [14C] acetate incorporation to DPS^20^
Adipose tissue	It is the major cholesterol storage in the body. In obesity over half of the total body cholesterol may reside within this tissue.^38^

In conclusion, statins mostly through reduction of cholesterol synthesis in hepatocytes alter the physiologic cholesterol balance between liver and extra-hepatic tissues; extra-hepatic tissues may synthesize additional cholesterol in order to reestablish this equilibrium.

Aside from *de novo* biosynthesis of cholesterol, further absorption of cholesterol in intestine can be regarded as a further mechanism of the cholesterol insufficiency compensation in the liver.

## Muscles and adverse effects of statins

Skeletal muscles comprise the major part of the body (∼50% of the total body Mass). They are the major site of metabolic activities and energy production.^39^ Therefore, myocytes may have less active metabolic pathways beyond the energy production and protein synthesis.

Thus, it is possible statins cause a severer cholesterol deficiency in skeletal muscle than other organs and tissues.

Regarding the remarkable role of cholesterol as a vital component of all cell membranes and also being precursor to numerous biological substances, cholesterol reduction by statins may influence the function of the all cellular membranes such as the sarcoplasmic reticulum and so affects the mechanisms involved in Ca^2+^ movements.^40^

In addition to cholesterol, mevalonate can be used for isoprenoids synthesis which are precursor to products such as ubiquinone, farnesol and geranylgeraniol which are used for prenylation of different proteins. Prenylation enables these proteins to bind to cell membrane and so activate several signaling pathways. Small “GTPases” and ‘lamins’ are the major targets of prenylation.^41^

Ras GTPases as prenylated proteins initiate a cascade of signaling events which culminate in up-regulation of cell growth. Rab GTPases as another target of prenylation, regulate organelle biogenesis and intracellular vesicular trafﬁcking.^42^

Also, inhibition of HMGCR reduces supply of ubiquinone as an important part of mitochondrial electron transport.^41^

In summary, the cholesterol synthesis reduction leads to disruption of the growth signaling pathways; reduction of the translocation of receptors to the plasma membrane and also reduction of the energy production. These effects induce apoptosis in myocytes^43^ as the most remarkable adverse effect of statins.

## Cholesterol and bile acids absorption in the intestine and therapeutic aims

**Figure 2:**
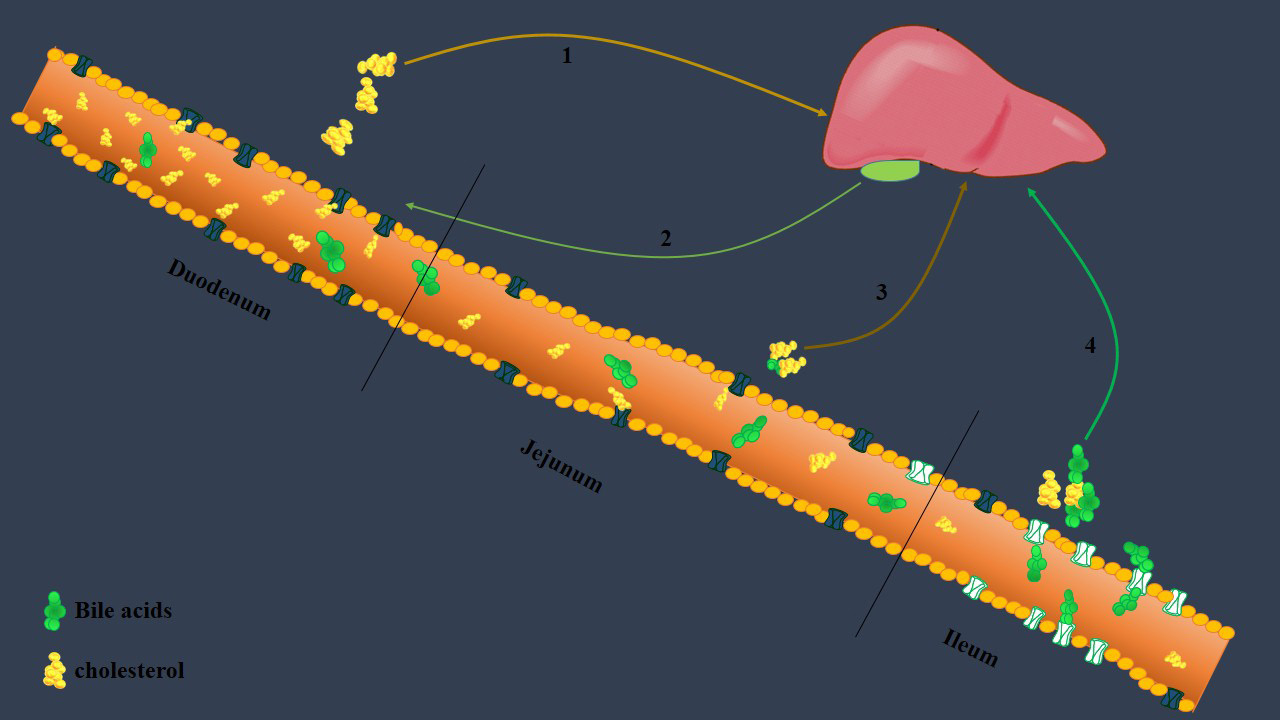
Three parts of small intestine have different roles in cholesterol and bile acids homeostasis. Duodenum is the main site of cholesterol absorption and ileum plays the major role in absorption of bile acids. Jejunum has an intermediate role in absorption of cholesterol and bile acids. Arrow 1 shows the higher volume of absorbed cholesterol in duodenum; arrow 2 shows the flow of bile from bile sac to duodenum; arrow 3 shows the moderate mild cholesterol and bile acids absorption in jejunum; arrow 4 shows the high volume of absorbed bile and moderate volume of cholesterol in ileum. After absorption of these compounds, they are transported into the liver and so enter into the intro-hepatical circulation. Therefore each part of small intestine has an important role in the cholesterol homeostasis the body.

Intestine plays a remarkable role in cholesterol metabolism through synthesis of cholesterol and absorption of cholesterol and bile acids in duodenum and ileum respectively (see Figure 2).

The cholesterol *de novo* biosynthesis versus dietary intake has been estimated as a ratio of ~70:30.^44^

Niemann-Pick C1Like 1 (NPC1L1) protein is the main proteins involved in the intestinal cholesterol absorption. The heterogeneous NPC1L1expression has the higher levels in the duodenum and jejunum, and the lower levels in the ileum.^45^

Also, 90%–95% of bile salts are reabsorbed in the ileum and these salts are transferred to the hepatocytes.^46^ In the liver, bile acids impose feedback-regulatory effects upon the synthesis of cholesterol and also their own synthesis. Bile acids have also been shown to alter the rate of cholesterogenesis in the small intestine.^47^ On the other hand, it has been emerged that excretion of bile acids in addition to the trans-intestinal cholesterol excretion (TICE) are the major pathway of cholesterol elimination from the body.^48^ Therefore, fluctuations of bile acids synthesis/absorption can affect cholesterol metabolism and vice versa as it has been shown that the reduced cholesterol levels in the liver by statins lead to further absorption of bile/diet cholesterol.^49^

Regarding the roles of ileum as the major site of bile acids absorption and a potent site of cholesterol synthesis in the body, it can be considered for treating of hypercholesterolemia.

Gastro-intestinal resections such as ileo-anal pouch, proctocolectomy and ileostomy may reduce the body cholesterol content^50^; therefore, resection of ileum, can be considered for more investigation in treating of patients with *hereditary* hypercholesterolemia.

In summary, different types of hyperlipidemia show different responses to statins. Understanding of cholesterol metabolism can be useful in treating of different types of hyperlipidemia.

## Conclusion

Cholesterol is an important bio-molecule in the body. The therapeutic effect of statin is not because of reduction of cholesterol; even it can be considered as one cause of the statin side effects, however it is because of the reduction of oxidized cholesterol through “reduction of the cholesterol half-life” in the body and subsequently reduction of the oxidized cholesterol content in circulation

It seems up-regulation of the HMGCR expression as a response to reduction of whole body cholesterol, cannot compensate the cholesterol deficiency in the liver. Thus extra-hepatic tissues such as gastrointestinal tract and adipose tissue gain the potential of synthesis of additional cholesterol to maintain cholesterol homeostasis in the body. Moreover, the intestinal absorption of cholesterol is the further mechanism for providing of required cholesterol in the body. Understanding of these mechanisms could be useful in disclosing of the mechanisms underlying the adverse effects of statins.

## Future work

In vitro targeting adipose tissue and ileum as the leading sites of cholesterol synthesis can be considered for more investigations as the therapeutic ways to improve the severe form of hypercholesterolemia.

In severe hereditary hypercholesterolemia like as familial hypercholesterolemia, surgical procedures such as “limited-ileostomy” (removing only limited portion of ileum, not as total ileostomy in the case of ileal cancers) can be considered for further investigations.

## Competing interests

The authors declare that they have no conflicts of interest.

## Ethical approval

Not applicable.

## References

[1] Wilson MD, Rudel LL (1994). Review of cholesterol absorption with emphasis on dietary and biliary cholesterol. J Lipid Res.

[2] Wang C-Y, Liu P-Y, Liao JK (2008). Pleiotropic effects of statin therapy: molecular mechanisms and clinical results. Trends Mol Med.

[3] Nebert DW, Russell DW (2002). Clinical importance of the cytochromes P450. Lancet.

[4] Lewis GF, Rader DJ (2005 24). New insights into the regulation of HDL metabolism and reverse cholesterol transport. Circ Res.

[5] Rigotti A, Miettinen HE, Krieger M (2003). The role of the high-density lipoprotein receptor SR-BI in the lipid metabolism of endocrine and other tissues. Endocr Rev.

[6] Bergstrom JD, Bostedor RG, Rew DJ, Geissler WM, Wright SD, Chao Y-S (1998). Hepatic responses to inhibition of 3-hydroxy-3-methylglutaryl-CoA reductase:a comparison of atorvastatin and simvastatin. Biochim Biophys Acta.

[7] Martin G, Duez H, Blanquart C, Berezowski V, Poulain P, Fruchart J-C (2001). Statin-induced inhibition of the Rho-signaling pathway activates PPARα and induces HDL apoA-I. J Clin Invest.

[8] Wong J, Quinn CM, Gelissen IC, Jessup W, Brown AJ (2008). The effect of statins on ABCA1 and ABCG1 expression in human macrophages is influenced by cellular cholesterol levels and extent of differentiation. Atherosclerosis.

[9] Argmann CA, Edwards JY, Sawyez CG, O’Neil CH, Hegele RA, Pickering JG (2005). Regulation of macrophage cholesterol efflux through hydroxymethylglutaryl-CoA reductase inhibition: a role for RhoA in ABCA1-mediated cholesterol efflux. J Biol Chem.

[10] Argani H, Ghorbani A, Rashtchizade N, Rahbaninobar M (2004). Effect of Lovastatin on Lipid peroxidation and total antioxidant concentrations in hemodialysis patients. Lipids Health Dis.

[11] Wong J, Quinn CM, Brown AJ (2004). Statins inhibit synthesis of an oxysterol ligand for the liver x receptor in human macrophages with consequences for cholesterol flux. Arterioscler Thromb Vasc Biol.

[12] Nagy L, Tontonoz P, Alvarez JG, Chen H, Evans RM (1998). Oxidized LDL regulates macrophage gene expression through ligand activation of PPARγ. Cell.

[13] Qiu G, Hill JS (2008). Atorvastatin inhibits ABCA1 expression and cholesterol efflux in THP-1 macrophages by an LXR-dependent pathway. J Cardiovasc Pharmacol.

[14] Sone H, Shimano H, Shu M, Nakakuki M, Takahashi A, Sakai M (2004). Statins downregulate ATP-binding-cassette transporter A1 gene expression in macrophages. Biochem Biophys Res Commun.

[15] Oram JF (2002). Molecular basis of cholesterol homeostasis:lessons from Tangier disease and ABCA1. Trends Mol Med.

[16] Cuchel M, Rader DJ (2006). Macrophage reverse cholesterol transport key to the regression of atherosclerosis?. Circulation.

[17] Li AC, Glass CK (2002). The macrophage foam cell as a target for therapeutic intervention. Nature Med.

[18] Kunjathoor VV, Febbraio M, Podrez EA, Moore KJ, Andersson L, Koehn S (2002). Scavenger receptors class AI/II and CD36 are the principal receptors responsible for the uptake of modified low density lipoprotein leading to lipid loading in macrophages. J Biol Chem.

[19] Argmann C, Sawyez C, McNeil C, Hegele R, Huff M (2003). Activation of peroxisome proliferator–activated receptor gamma and retinoid x receptor results in net depletion of cellular cholesteryl esters in macrophages exposed to oxidized lipoproteins. Arterioscler Thromb Vasc Biol.

[20] Jeske DJ, Dietschy JM (1980). Regulation of rates of cholesterol synthesis in vivo in the liver and carcass of the rat measured using [3H] water. J lipid Res.

[21] Song G, Liu J, Zhao Z, Yu Y, Tian H, Yao S (2011). Simvastatin reduces atherogenesis and promotes the expression of hepatic genes associated with reverse cholesterol transport in apoE-knockout mice fed high-fat diet. Lipids Health Dis.

[22] Eriksson M, Carlson LA, Miettinen TA, Angelin B (1999). Stimulation of fecal steroid excretion after infusion of recombinant proapolipoprotein AI potential reverse cholesterol transport in humans. Circulation.

[23] Andrikoula M, McDowell I (2008). The contribution of ApoB and ApoA1 measurements to cardiovascular risk assessment. Diabetes Obes Metab.

[24] Halloran L, Schwartz C, Vlahcevic Z, Nisman R, Swell L (1978). Evidence for high-density lipoprotein-free cholesterol as the primary precursor for bile-acid synthesis in man. Surgery.

[25] Chapman M (2004). Are the effects of statins on HDL-cholesterol clinically relevant?. Eur Heart J Suppl.

[26] Wood C, Jalil MNH, McLaren I, Yong BC, Ariffin A, McNeil PH (1984). Carnitine long-chain acyltransferase and oxidation of palmitate, palmitoyl coenzyme A and palmitoylcarnitine by pea mitochondria preparations. Planta.

[27] Javitt N (1994). Bile acid synthesis from cholesterol:regulatory and auxiliary pathways. FASEB J.

[28] Lampl Y. Serum Lipids and Statin Treatment During Acute Stroke. INTECH; 2012.

[29] Gazzerro P, Proto MC, Gangemi G, Malfitano AM, Ciaglia E, Pisanti S (2012). Pharmacological actions of statins:a critical appraisal in the management of cancer. Pharm Rev.

[30] Rader DJ (2003). Regulation of reverse cholesterol transport and clinical implications. Am J Cardiol.

[31] Yoshida M, Harada N, Yoshida K, Nakagawa T, Shimohata T, Mawatari K (2010). High density lipoprotein inhibits the activation of sterol regulatory element-binding protein-1 in cultured cells. FEBS Lett.

[32] Kuipers F. New insights in the role of the intestine in reverse cholesterol transport. In: Junien JL, ‎Staels B. Nuclear Receptors as Molecular Targets for Cardiometabolic and Central Nervous System Diseases IOS Press; 2008.

[33] Siperstein MD, Guest MJ (1960). Studies on the site of the feedback control of cholesterol synthesis. J Clin Inves.

[34] Parker RA, Clark RW, Sit S-Y, Lanier TL, Grosso RA, Wright J (1990). Selective inhibition of cholesterol synthesis in liver versus extrahepatic tissues by HMG-CoA reductase inhibitors. J Lipid Res.

[35] Dietschy JM, Wilson JD (1968). Cholesterol synthesis in the squirrel monkey:relative rates of synthesis in various tissues and mechanisms of control. J Clin Invest.

[36] Dietschy JM, Gamel WG (1971). Cholesterol synthesis in the intestine of man:regional differences and control mechanisms. J Clin Invest.

[37] Dietschy JM, Siperstein MD (1967). Effect of cholesterol feeding and fasting on sterol synthesis in seventeen tissues of the rat. J Lipid Res.

[38] Krause BR, Hartman AD (1984). Adipose tissue and cholesterol metabolism. J Lipid Res.

[39] Nader GA (2005). Molecular determinants of skeletal muscle mass:getting the “AKT” together. Int J Biochem Cell Biol.

[40] Pierno S, De Luca A, Liantonio A, Camerino C, Camerino DC (1999). Effects of HMG-CoA reductase inhibitors on excitation–contraction coupling of rat skeletal muscle. Eur J Pharmacol.

[41] Abd TT, Jacobson TA (2011). Statin-induced myopathy:a review and update. Expert Opin Drug Saf.

[42] Westwood FR, Bigley A, Randall K, Marsden AM, Scott RC (2005). Statin-induced muscle necrosis in the rat:distribution, development, and fibre selectivity. Toxicol Pathol.

[43] Vaklavas C, Chatzizisis YS, Ziakas A, Zamboulis C, Giannoglou GD (2009). Molecular basis of statin-associated myopathy. Atherosclerosis.

[44] Ikonen E (2008). Cellular cholesterol trafficking and compartmentalization. Nature Rev Mol Cell Biol.

[45] Ninomiya H (2010). Niemann-Pick C1-Like 1: A Key Player in Intestinal Cholesterol Absorption. Yonago Acta Med.

[46] Jansen PL, Faber KN. Metabolism of bile acids. In: Juan Rodés J, Benhamou JP, Blei A, Reichen J, Rizzetto M, eds. The Textbook of Hepatology: From Basic Science to Clinical Practice. 3rd ed. Wiley; 2007.

[47] Dietschy JM (1968). Mechanisms for the intestinal absorption of bile acids. J Lipid Res.

[48] Van der Velde AE, Brufau G, Groen AK (2010). Transintestinal cholesterol efflux. Curr Opin Lipidol.

[49] Santosa S, Varady KA, AbuMweis S, Jones PJ (2007). Physiological and therapeutic factors affecting cholesterol metabolism: does a reciprocal relationship between cholesterol absorption and synthesis really exist?. Life Sci.

[50] Kostapanos MS, Milionis HJ, Elisaf MS (2008). An overview of the extra-lipid effects of rosuvastatin. J Cardiovasc Pharmacol Ther.

